# A modified two-compartment model for measurement of renal function using dynamic contrast-enhanced computed tomography

**DOI:** 10.1371/journal.pone.0219605

**Published:** 2019-07-10

**Authors:** Kai Jiang, Christopher M. Ferguson, Abdelrhman Abumoawad, Ahmed Saad, Stephen C. Textor, Lilach O. Lerman

**Affiliations:** Division of Nephrology and Hypertension, Mayo Clinic, Rochester, Minnesota, United States of America; University Medical Center Utrecht, NETHERLANDS

## Abstract

**Objectives:**

To validate and adapt a modified two-compartment model, originally developed for magnetic resonance imaging, for measuring human single-kidney glomerular filtration rate (GFR) and perfusion using dynamic contrast-enhanced computed tomography (DCE-CT).

**Methods:**

This prospective study was approved by the institutional review board, and written informed consent was obtained from all patients. Thirty-eight patients with essential hypertension (EH, n = 13) or atherosclerotic renal artery stenosis (ARAS, n = 25) underwent renal DCE-CT for GFR and perfusion measurement using a modified two-compartment model. Iothalamate clearance was used to measure reference total GFR, which was apportioned into single-kidney GFR by renal blood flow. Renal perfusion was also calculated using a conventional deconvolution algorithm. Validation of GFR and perfusion and inter-observer reproducibility, were conducted by using the Pearson correlation and Bland-Altman analysis.

**Results:**

Both the two-compartment model and iothalamate clearance detected in ARAS patients lower GFR in the stenotic compared to the contralateral and EH kidneys. GFRs measured by DCE-CT and iothalamate clearance showed a close match (r = 0.94, P<0.001, and mean difference 2.5±12.2mL/min). Inter-observer bias and variation in model-derived GFR (r = 0.97, P<0.001; mean difference, 0.3±7.7mL/min) were minimal. Renal perfusion by deconvolution agreed well with that by the compartment model when the blood transit delay from abdominal aorta to kidney was negligible.

**Conclusion:**

The proposed two-compartment model faithfully depicts contrast dynamics using DCE-CT and may provide a reliable tool for measuring human single-kidney GFR and perfusion.

## Introduction

Glomerular filtration rate (GFR) is a fundamental index of kidney function. Serum clearance of endogenous or exogenous markers is typically used to estimate GFR [1], but cannot be used to evaluate single-kidney function, which is important to assess in asymmetric renal diseases, such as renal artery stenosis, ureteral obstruction, or renal tumors. Dynamic contrast-enhanced computed tomography (DCE-CT) and magnetic resonance imaging (DCE-MRI) have emerged as useful tools for measuring single-kidney GFR as well as renal perfusion.

Assessment of renal hemodynamics and function using either DCE-CT or DCE-MRI usually involves application of mathematical models based on several assumptions describing contrast media kinetics and their relationship with signal intensity. Different methods have been proposed for GFR measurement from DCE-CT, including the Patlak method [2] and its derivatives [3–6]. However, this method is only valid when the fitting encompasses data after the vascular peak and before contrast outflow from the selected region of interest (ROI) [5,7]. For patients with altered tubular transit time, the selection of fitting duration might be problematic, and may cause inaccurate GFR estimation. In addition, the Patlak method does not offer important renal parameters such as perfusion or tubular transit times. More advanced models, including the gamma-variate model [8,9], have been developed to resolve this issue, but require careful selection of ROIs, and may overestimate GFR [9]. Hence, application of robust models could facilitate acquisition of DCE-CT-derived functional data.

For measurement of single-kidney GFR using DCE-MRI, elegant two- [10,11] and three- [12,13] compartment models have been proposed to delineate gadolinium kinetics in kidneys, but have been shown to underestimate GFR [10,12–14]. Recently, a novel modified two-compartment model was developed for DCE-MRI and shown to offer accurate estimation of single-kidney GFR as well as perfusion in mice [15]. Compared to other existing two-compartment models [11,16], the major advantage of this novel model is incorporation of the contrast outflow dynamics, thereby inherently accounting for the tubular transit delay. The incorporation of contrast outflow curve in our model fitting allows for more reliable estimation of kidney function, because tubular flow rates (and thus fitting ranges) may be altered under pathological conditions. However, to date, this model has not been implemented with any other imaging modalities, contrast agents, or species. Therefore, the specificity of this model for gadolinium kinetics in the kidney and its relationship with MR signal intensity, or whether it allows single-kidney GFR estimation in humans, all remain unknown.

We hypothesized that our modified two-compartment model originally developed for MRI would robustly depict the filtration process of iodinated contrast media in human kidneys, as detected using DCE-CT. Single-kidney GFR and perfusion by DCE-CT were validated against those calculated by iothalamate clearance and a conventional deconvolution algorithm, respectively. Inter-observer reproducibility of image analysis using the proposed model was assessed. In a simulation study we also evaluated the need for introducing in the model a blood transit delay from abdominal aorta to kidney, which has been inconsistently applied in previous studies [11,13,14]. Furthermore, because GFR measurement using DCE-CT is limited by the use of ionizing radiation [17], we also took the opportunity to estimate the minimally required CT dynamic imaging time to meet the ALARA (‘as low as reasonably achievable’) principle.

## Materials and methods

### Patients

The prospective study was approved by the institutional review board and Health Insurance Portability and Accountability Act compliant. Prior to clinical examinations, written informed consent was obtained from each patient. A total of thirty-eight hypertensive patients with either essential hypertension (EH, n = 13) or renovascular hypertension secondary to atherosclerotic renal artery stenosis (ARAS, n = 25) were studied. ARAS was diagnosed given cross-sectional obstruction of more than 60% in renal artery using CT or blood velocity larger than 30 cm/sec using Doppler ultrasonography. Five ARAS patients had bilateral stenosis, resulting in a total of 30 stenotic and 20 contralateral kidneys. Only patients with serum creatinine level below 2.5 mg/dL were included in this study, to avoid potential nephrotoxicity of CT contrast media. For uniformity, all patients were treated with angiotensin-converting enzyme inhibitors or angiotensin receptor blockers anti-hypertensive therapy during inpatient studies in the Clinical Research and Trials Unit at St. Mary’s Hospital (Rochester, MN) while ingesting a fixed sodium diet (150 mEq/d). All patients underwent GFR measurement using firstly iothalamate clearance, and then CT two days later.

### Renal function and blood pressure

As a reference, GFR was measured from plasma clearance of iothalamate meglumine (Conray, Mallinckrodt Pharmaceuticals, St Louis, MO), which has been widely adopted for GFR measurement in clinic trials [18]. For each patient, 0.5 mL iothalamate was subcutaneously injected, followed by three 30-minute blood sampling periods after oral hydration at 20 mL/kg. Total GFR was calculated from the time course of blood iothalamate concentration, as described previously [19,20]. Then the total GFR was apportioned by model-derived renal blood flow (RBF) to achieve the single-kidney GFR. Blood pressure was measured using automated oscillometric recording. To assess the serum creatinine level, blood samples were obtained from the inferior venal cava, as described previously [21]. Estimated GFR was quantified using the Modification of Diet in Renal Disease equation.

### CT studies

CT studies were performed using a dual-source 64-slice helical multidetector CT scanner (Somatom Definition; Siemens, Forchheim, Germany). Imaging parameters were selected in line with technical prerequisites of modern state-of-the-art CT perfusion imaging [22] as described previously [17]. Briefly, following a central venous injection of Iopamidol-370 (0.5 mL/kg), dynamic CT imaging was conducted during coached intermittent respiratory suspension. A total of 45 acquisitions were acquired, including 35 scans distributed into three ≤20-sec breath-holds, and 10 additional scans with intermittent short breath-holds. The rotation time of the scanner was 0.5 second. The temporal resolution was 1 sec/scan during the vascular phase to capture the rapid contrast dynamics and 8 sec/scan during the tubular phase, during which contrast dynamics changed more slowly. Depending on patients’ body weight, tube voltage was set at 80, 100, or 120 kVp and current at 160 or 250 mAs with 24×1.2 collimation and 0 table feed. Four slices (7.2-mm thick) localized at kidney hilum were scanned in approximately 145 sec to follow the contrast bolus throughout the renal tubular system. After fifteen minutes, a helical scan was performed following another contrast injection to enhance the corticomedullary contrast for determination of cortical and medullary volumes. Imaging parameters were: tube voltage 100 or 120 kVp, tube current from 72 to 350 mAs, scan length from 25 to 40 cm, spatial resolution, 0.59×0.59×5 mm^3^, and spiral pitch factor 1.2. Image reconstruction for the DCE-CT and volumetric scans was performed using a B35s and B40f kernel, respectively.

### Compartment model

**Fig 1:**
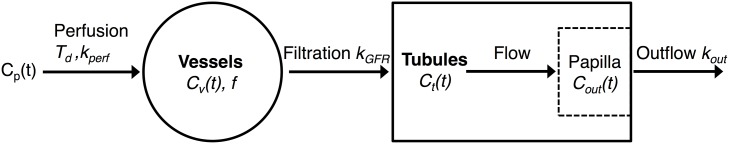
The modified two-compartment model. The kidney is simplified as a combination of blood vessels and renal tubules. Plasma flow carrying the iodinated contrast enters the kidney vessels and is filtered by the glomeruli, after which leaves the kidney from the papilla following tubular flow. *C*_*p*_*(t)* is the arterial input function; *T*_*d*_ is the blood transit delay; *k*_*perf*_ is the perfusion rate constant; *C*_*v*_*(t)* is the iodine concentration in renal vessels; *f* is the volume fraction of renal vascular space; *k*_*GFR*_ is the normalized GFR; *C*_*t*_*(t)* is the iodine concentration in the tubules distributed over the entire kidney; *C*_*out*_*(t)* is the iodine concentration in the papilla; *k*_*out*_ is the iodine outflow rate constant.

**Fig 2:**
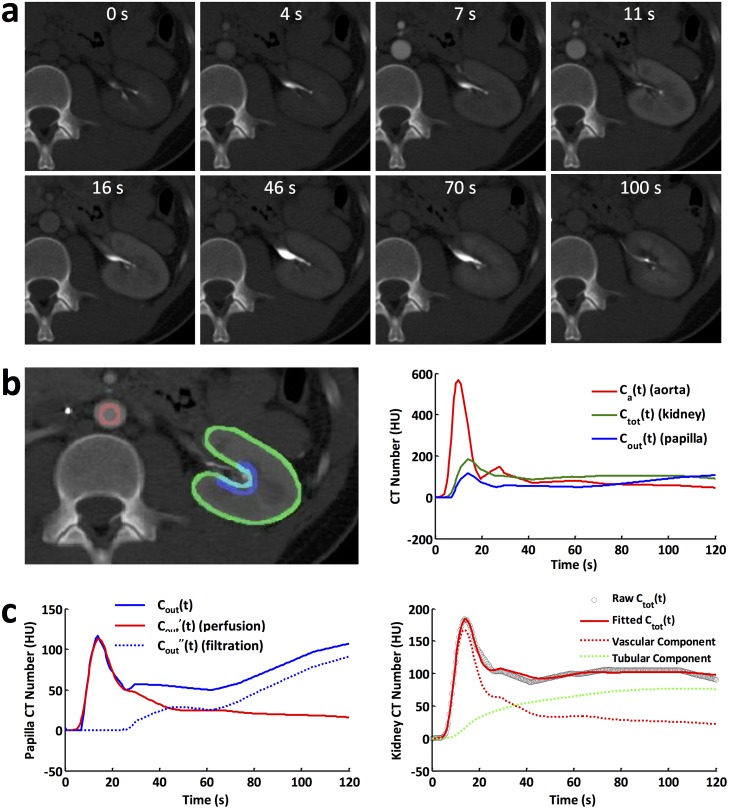
DCE-CT and model fitting. (a) Representative CT images acquired at baseline, and 4, 7, 11, 16, 46, 70, 100 sec post-contrast injection. (b) Three ROIs (left) manually traced on abdominal aorta (red), renal parenchyma (green), and papilla (blue), and their respective time attenuation curves (right). (c) Time attenuation curve in renal papilla decomposed into perfusion and filtration components (left), and model fitting to the time attenuation curve in the renal parenchyma, as well as the fitted vascular and tubular components.

To calculate single-kidney GFR and perfusion, we implemented a model originally developed to measure murine renal function from gadolinium dynamics captured using MRI. As shown in Fig 1, the kidney is simplified as a combination of blood vessels and renal tubules. Plasma flow carrying the iodinated contrast with the concentration of *C*_*p*_*(t)* enters the kidney vessels and is filtered by the glomeruli, after which leaves the kidney from the papilla following tubular flow. The iodine concentrations in renal vessels, tubules distributed over the entire kidney, and papilla are represented by *C*_*v*_*(t)*, *C*_*t*_*(t)*, and *C*_*out*_*(t)*, respectively. The blood transit delay from abdominal aorta to kidney vessels is denoted as *T*_*d*_. Notably, such delay was negligible in murine kidneys, but may not be in human kidneys, given the substantial difference in heart rate and blood kinetics [23,24]. The volume fraction of renal vessels is *f*. The perfusion, filtration, and efflux rate constants are *k*_*perf*_, *k*_*GFR*_, and *k*_*out*_, respectively. The dynamics of *C*_*v*_*(t)* and *C*_*t*_*(t)* can be described by the following first-order differential equations:

$$\frac{dC_{v}(t)}{dt}=k_{perf}(C_{p}(t-T_{d})-C_{v}(t))$$ (1)

$$\frac{dC_{t}(t)}{dt}=k_{GFR}C_{v}(t)-k_{out}C_{out}^{''}(t)$$ (2)

where *C*_*p*_*(t)* is the arterial input function and calculated as *C*_*a*_*(t)/(1-Hct)*, where *C*_*a*_*(t)* is the iodine concentration in inflow blood from abdominal aorta and Hct the assumed hematocrit (45%). The total iodine concentration in the renal papilla (*C*_*out*_*(t)*) comprises of two components, one from perfusion (*C*_*out*_^*’*^*(t)*) and the other from tubular transit (*C*_*out*_^***”***^*(t)*). The perfusion component (*C*_*out*_^*’*^*(t)*) can be derived from *C*_*p*_*(t)* and the papilla perfusion rate constant, which can be fitted from the early tubular phase (30 to 40s) prior to contrast arrival using Eq 1. Then *C*_*out*_^***”***^*(t)* was achieved by subtracting *C*_*out*_*(t)* from *C*_*out*_^*’*^ *(t)* (Fig 2c, left). Inclusion of the papilla output curve *C*_*out*_^***”***^*(t)* enables timing of contrast outflow and accurate delineation of the kidney output curve, both of which are important for accurate model fitting. This approach has been found to be critical in mouse kidneys [15] and necessary in human kidneys.

The total iodine concentration in the kidney can be calculated as *C*_*tot*_*(t)* = *C*_*v*_*(t)*·*f* + *C*_*t*_*(t)*. A total of five unknown parameters, *k*_*perf*_, *T*_*d*_, *f*, *k*_*GFR*_, and *k*_*out*_, were fitted, after which the single-kidney GFR (mL/min) and perfusion were calculated as:

$$GFR\;(\frac{mL}{min})=k_{GFR}(s^{-1})∙V(mL)∙60(\frac{s}{min})$$ (3)

$$Perfusion\;(\frac{ml}{100g∙min})=k_{perf}(s^{-1})∙f∙60(\frac{s}{min})∙\frac{100ml}{100ml}/(1-Hct)∙\sigma (\frac{ml}{g})$$ (4)

where *V* is 3D CT-measured renal volume and σ represents the blood/tissue partition coefficient for water, which is 0.9 [25,26]. Renal blood flow was subsequently quantified as the product of perfusion and volume.

### Image analysis

Renal volumes were measured from the helical CT images using Analyze (Biomedical Imaging Resource, Mayo Clinic, MN). Kidneys were manually selected on CT images with sinus fat and major vessels excluded.

The DCE-CT images were post-processed using a Matlab (Mathworks, Natick, MA) module developed in-house. For each kidney, the slice with the largest renal cross-sectional area was analyzed. Images with in-plane motion were aligned using rigid registration. Specifically, a kidney mask was defined on one image without motion, after which the kidney edge was detected and propagated to other images. Then, on images showing in-plane motion, the kidney mask was manually shifted to align with the kidney. Images with off-plane motion were excluded. ROIs were manually selected on the abdominal aorta, renal parenchyma, and papilla, a 3-pixel-wide strip of the renal parenchyma where filtrate outflow converges. The ROI for the papilla was semiautomatically selected to facilitate image analysis as well as reduce inter-operator variability. Specifically, the kidney mask edge was detected and dilated using a 6×6 kernel. Then an approximate region containing the renal pelvis was manually selected, and the overlapping area of this region and the dilated kidney mask edge was set as the ROI for measurement of the contrast output function. These were used for measurement of arterial input function (*C*_*p*_*(t)*), contrast dynamics in kidney (*C*_*tot*_*(t)*), and contrast output function (*C*_*out*_*(t)*), respectively (Fig 2b, left). A linear interpolation was used to equalize the distribution of sampled data points with an interval of 0.5 sec. The increase in CT signal from baseline was used to represent the concentration of iodine [27]. Due to motion artifacts in later scans, only time attenuation curves from 0 to 120 sec were used in the model fitting. The model fitting was performed by minimizing the sum of squares of fitting errors using the Levenberg–Marquardt algorithm. In order to test the goodness of fit, the coefficients of multiple correlation for the EH, stenotic, and contralateral kidneys were calculated. After measuring single-kidney GFRs, total GFR was quantified as the sum.

To assess the reliability of the compartment model in perfusion measurement, renal perfusion maps were additionally generated using a truncated singular value decomposition-based deconvolution algorithm [28] implemented in the OsiriX Lite software (version 10.0, Pixmeo, Geneva, Switzerland) [29]. CT images capturing the first pass within 20 sec after bolus injection were used in order to minimize the influence of renal filtration on perfusion measurement [30]. Then reference renal perfusion was quantified from the perfusion map using the same ROI defined in the model analysis. Since ignoring blood transit delay in the deconvolution algorithm is known to underestimate renal perfusion [28], the patient kidneys were divided into two groups by model-derived transit delay using a cutoff value of 1 sec. Then comparison of renal perfusion measured by the model and deconvolution was conducted for each group individually.

To determine the inter-observer reproducibility of ROI placement and subsequent model fitting of renal functional parameters, GFR and RBF measured by two independent operators in all EH and ARAS patients were compared using both correlation and Bland-Altman analyses.

#### Simulation studies

To demonstrate the impact of neglecting the blood transit delay (*T*_*d*_) from abdominal aorta to renal vasculature, a separate curve fitting was conducted on all kidneys with T_d_ fixed at zero. Then the measured renal perfusion and normalized GFR were compared against those fitted with T_d_ as an unknown parameter. Additionally, the necessity of including T_d_ in the model fitting was tested with various fitting ranges and imaging temporal resolutions. To investigate the impact of the fitting range, renal perfusion and GFR with and without T_d_ as an unknown parameter were quantified with different fitting ranges from 50 to 120 sec with 5-sec incremental steps, with the temporal resolution fixed at 0.5 s/scan. Similarly, the impact of temporal resolution was investigated by fitting resampled CT dynamic curves with the temporal resolution ranging from 0.5 to 3.5 s/scan at 0.2 s/scan incremental steps, with the fitting range maintained at 120 s. Then renal perfusion and GFR ratios were calculated by normalizing estimated renal parameters without T_d_ as an unknown parameter by those with T_d_ as an unknown parameter.

To investigate the impact of fitting range on the accuracy of perfusion and GFR estimation, Monte-Carlo simulation was performed on all kidneys by varying the fitting range from the initial 50 to 120 sec with 5-sec incremental steps. Then the measured renal perfusion and GFR with different fitting ranges were normalized by those measured with the fitting range at 120 sec. The averaged value and standard deviation of normalized perfusion and GFR at different fitting ranges were calculated for comparison.

#### Statistical analysis

All statistical analysis was performed using JMP 13.0 (SAS Institute, Cary, NC). The Shapiro-Wilk test was used to test the normality of data. Data with normal distribution was expressed as means ± standard deviations and non-normal distribution as medians with interquartile ranges. For normally distributed data, one-way analysis of variance with student’s unpaired or paired t-test was performed for comparisons among groups. Non-normally distributed data were compared using the Kruskal-Wallis test followed by the Wilcoxon signed-rank test or Mann-Whitney U test. For validation of the DCE-CT measured GFR and renal perfusion, and evaluation of inter-observer reproducibility of image analysis using the proposed model, Pearson correlation and Bland-Altman analysis were performed. Statistical significance was judged with P value less than 0.05.

## Results

### Baseline characteristics of patients

All relevant data underlying the statistics as well as the extracted time attenuation curves from the CT images are shown in S1 Dataset in Supporting Information. As shown in Table 1, EH and ARAS patients had similar percentage of males, age, body mass index, and number of antihypertensive drugs. No difference in systolic, diastolic, and mean arterial pressure was observed between EH and ARAS patients. However, elevated serum creatinine level in ARAS patients indicated their impaired renal function. Both estimated and iothalamate clearance-measured total GFR were lower in ARAS patients.

**Table 1:** Baseline characteristics of patients.

	EH (n = 13)	ARAS (n = 25)	P Value
No. of males	7 (54%)	15 (60%)	0.716
Age	67.0 (62.0–71.0)	70.0 (58.0–74.0)	0.320
No. of antihypertensive drugs	2 (2–3)	3 (3–4)	0.168
BMI (kg/m^2^)	28.5 ± 3.7	27.6 ± 4.0	0.872
Blood pressure (mmHg)			
Systolic	129.6 ± 16.1	141.2 ± 20.4	0.083
Diastolic	70.8 ± 9.9	70.1 ± 11.1	0.844
MAP	90.4 ± 9.7	93.8 ± 11.5	0.370
Serum creatinine level (mg/dL)	0.90 ± 0.21	1.28 ± 0.40	0.003
Estimated GFR (mL/min per 1.73 m^2^)	77.6 (67.0–87.0)	53.8 (42.6–65.4)	0.007
GFR by iothalamate clearance (mL/min)	89.2 (81.0–99.5)	60.1 (47.0–90.3)	0.007

Note: Data are expressed in mean ± standard deviation or median with interquartile ranges in parentheses, as appropriate. EH = essential hypertension; ARAS = atherosclerotic renal artery stenosis; BMI = body mass index; MAP = mean arterial pressure; GFR = glomerular filtration rate.

### Model fitting of DCE-CT data

Representative CT images of one EH kidney at baseline and different time intervals after contrast injection are shown in Fig 2a. The time attenuation curves in abdominal aorta (red), kidney parenchyma (green), and papilla (blue) were measured from manually traced ROIs (Fig 2b). The arterial input function shows an initial sharp peak, a second low peak due to contrast recirculation, and a gradual decrease thereafter. The iodine concentration in renal parenchyma is characterized by an initial increase due to perfusion, a slow rise because of glomerular filtration, and then a slight decrease as a result of outflow. Similar initial increase in iodine concentration is observed in the papilla because of perfusion, followed by a steady state during tubular transit, and subsequent rapid increase as a result of iodine inflow.

The DCE and volumetric CT scans yielded dose-length product values of 913.3 ± 294.8 and 339.6 ± 85.1 mGy·cm, respectively. Using a conversion factor of 0.0153 for the abdomen [31], the effective dose values of the DCE and volumetric scans were 13.8 ± 4.7 and 5.2 ± 1.3 mSv, respectively. Thus, the total effective dose of our CT scans is 19.0 ± 5.1 mSv, which is comparable or lower than other the dose of similar multiphasic abdominal CT scans [32].

The decomposition of papilla iodine concentration (*C*_*out*_*(t)*) to the perfusion (*C*_*out*_^*’*^*(t)*) and filtration (*C*_*out*_^*”*^*(t)*) components are shown in Fig 2c (left). The experimentally-acquired raw and model-fitted time attenuation curves for the kidney parenchyma as well as its vascular and tubular components are shown in Fig 2c (right). The fitted curve showed an excellent agreement with the raw data, suggesting that the proposed compartment model faithfully depicts the kidney filtration process in human kidneys. In addition, the quantified coefficients of multiple correlation for the EH (0.94 ± 0.06), stenotic (0.93 ± 0.12), and contralateral (0.95 ± 0.07) kidneys were all close to 1, further supporting the reliability of model fitting.

Shown in Table 2 are the CT-measured kidney volume and the model-derived parameters. Stenotic kidneys had smaller volume (P<0.001), as compared to both EH and contralateral kidneys. While *k*_*perf*_, *T*_*d*_, and *k*_*out*_ were unchanged, *f* (stenotic, P = 0.010; contralateral, P = 0.005) and *k*_*GFR*_ (stenotic, P<0.001; contralateral, P = 0.038) fell significantly in both the stenotic and contralateral kidneys, as compared to EH kidneys.

**Table 2:** Kidney volume and model-fitted parameters.

	EH Kidney	Stenotic Kidney	Contralateral Kidney
Volume (mL)	148 ± 33	86 ± 44*	170 ± 50^†^
*k*_*perf*_ (s^-1^)	0.15 (0.11–0.19)	0.12 (0.09–0.17)	0.17 (0.11–0.18)
*T*_*d*_ (s)	0.68 (0.34–1.16)	0.58 (0.05–1.35)	1.02 (0.47–1.57)
*f*	0.22 ± 0.04	0.19 ± 0.06*	0.18 ± 0.04*
*k*_*GFR*_ (s^-1^)	0.0048 ± 0.0009	0.0035 ± 0.0013*	0.0041 ± 0.0011*
*k*_*out*_ (s^-1^)	0.004 (0.003–0.011)	0.005 (0.003–0.012)	0.007 (0.002–0.009)

Note: Data are expressed in mean ± standard deviation or median with interquartile ranges in parentheses, as appropriate.

*P<0.05 compared to EH kidney,

^†^P<0.05 compared to stenotic kidney.

EH = essential hypertension.

### GFR by CT and iothalamate clearance

**Fig 3:**
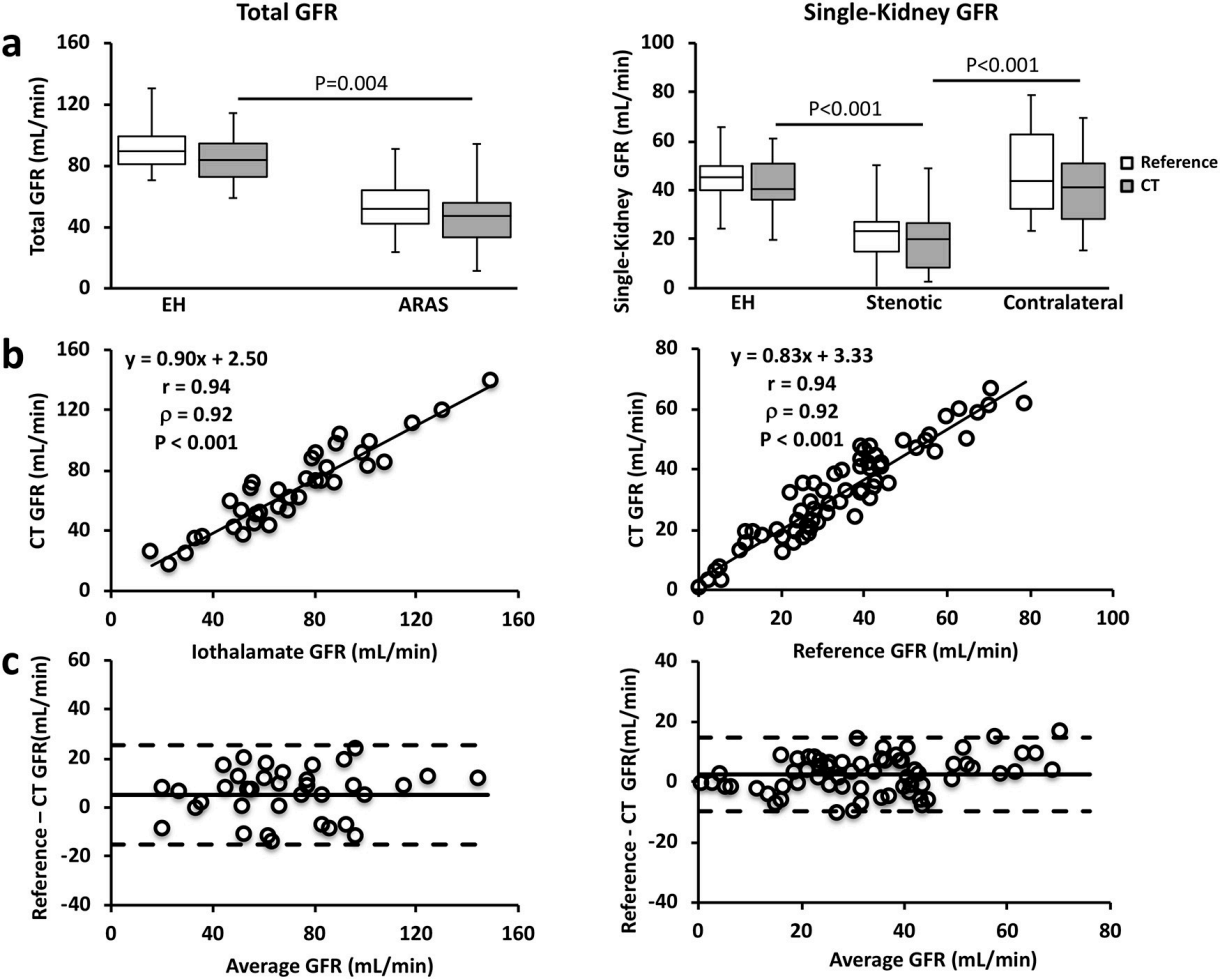
Validation of model-derived GFR by DCE-CT using iothalamate clearance. (a) Total (left) and single-kidney (right) GFRs measured by DCE-CT and iothalamate clearance in EH and ARAS patients. (b) Correlation between DCE-CT and iothamate clearance in measuring total (left) and single-kidney (right) GFRs. (c) Bland-Altman analysis of total (left) and single-kidney (right) GFR measurements by DCE-CT and iothalamate clearance.

In both EH and ARAS patients, total kidney GFRs by DCE-CT and iothalamate clearance were comparable (Fig 3a, left), and well correlated (Fig 3b, left, Pearson correlation coefficient r = 0.94, P<0.001). A good agreement between them (mean difference of 5.0±20.3 mL/min) was also observed in the Bland-Altman analysis (Fig 3c, left). The stenotic kidneys had lower CT-measured GFR (20.0 (8.4–26.6) mL/min, Fig 3a, right), as compared to the EH (40.4 (36.1–50.9) mL/min, P<0.001) and contralateral kidneys (41.2 (28.3–50.9) mL/min, P<0.001). The GFRs in the contralateral and EH kidneys were similar. Single-kidney GFRs by both methods were also similar (Fig 3a, right) and showed a good correlation (Fig 3b, right, r = 0.94, P<0.001). A good agreement was also observed in the Bland-Altman analysis (Fig 3c, right, mean difference, 2.5±12.2 mL/min).

### Renal perfusion and blood flow

**Fig 4:**
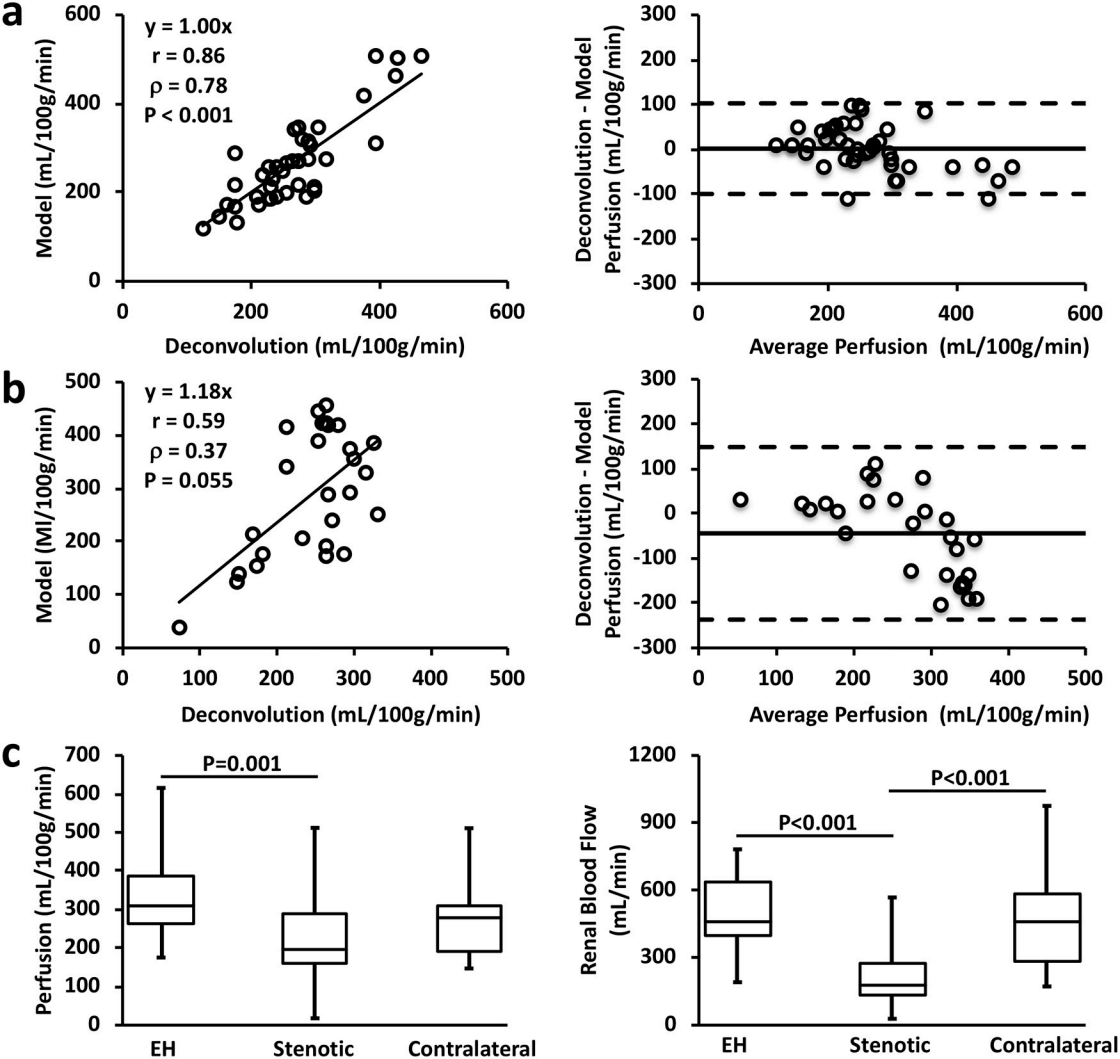
Renal perfusion and blood flow by DCE-CT. (a-b) Correlation (left) and Bland-Altman (right) analyses of renal perfusion derived from the deconvolution algorithm and model fitting in kidneys with blood transit delay below (a) and above (b) 1 sec. (c) Model-derived renal perfusion (left) and calculated renal blood flow (right) in all EH, stenotic, and contralateral kidneys.

When the blood transit delay from abdominal aorta to kidney was under 1 sec, a good agreement in renal perfusion by the compartment model and deconvolution was observed, as shown by the correlation (Fig 4a left, r = 0.86, P<0.001) and Bland-Altman (Fig 4a right, mean difference, 1.4±101.2 mL/100g/min) analyses. However, with delay exceeding 1 sec, the deconvolution algorithm yielded smaller perfusion values than the compartment model, especially at high perfusion rates (Fig 4b). These observations are consistent with previous findings that this deconvolution approach underestimated perfusion values by ignoring the blood transit delay, which was accentuated with longer delays and high perfusion [28].

The DCE-CT measured renal perfusion and blood flow are shown in Fig 4c. The ARAS stenotic kidneys showed a 36.8% decrease in perfusion compared to the EH kidneys (Fig 4c left, 194.8 (162.0–290.2) vs. 308.1 (261.6–385.7) mL/100g/min, P = 0.001). No change was observed in perfusion of the contralateral kidneys (280.8 (191.8–310.6) mL/100g/min) compared to the EH kidneys. The calculated RBF in the stenotic kidneys (Fig 4c right, 182.0 (130.0–274.1) mL/min) was found decreased by 60.7% and 60.1%, as compared to the EH (463.3 (398.1–639.0) mL/min, P<0.001) and contralateral (456.2 (282.3–579.6) mL/min, P<0.001) kidneys, respectively.

**Fig 5:**
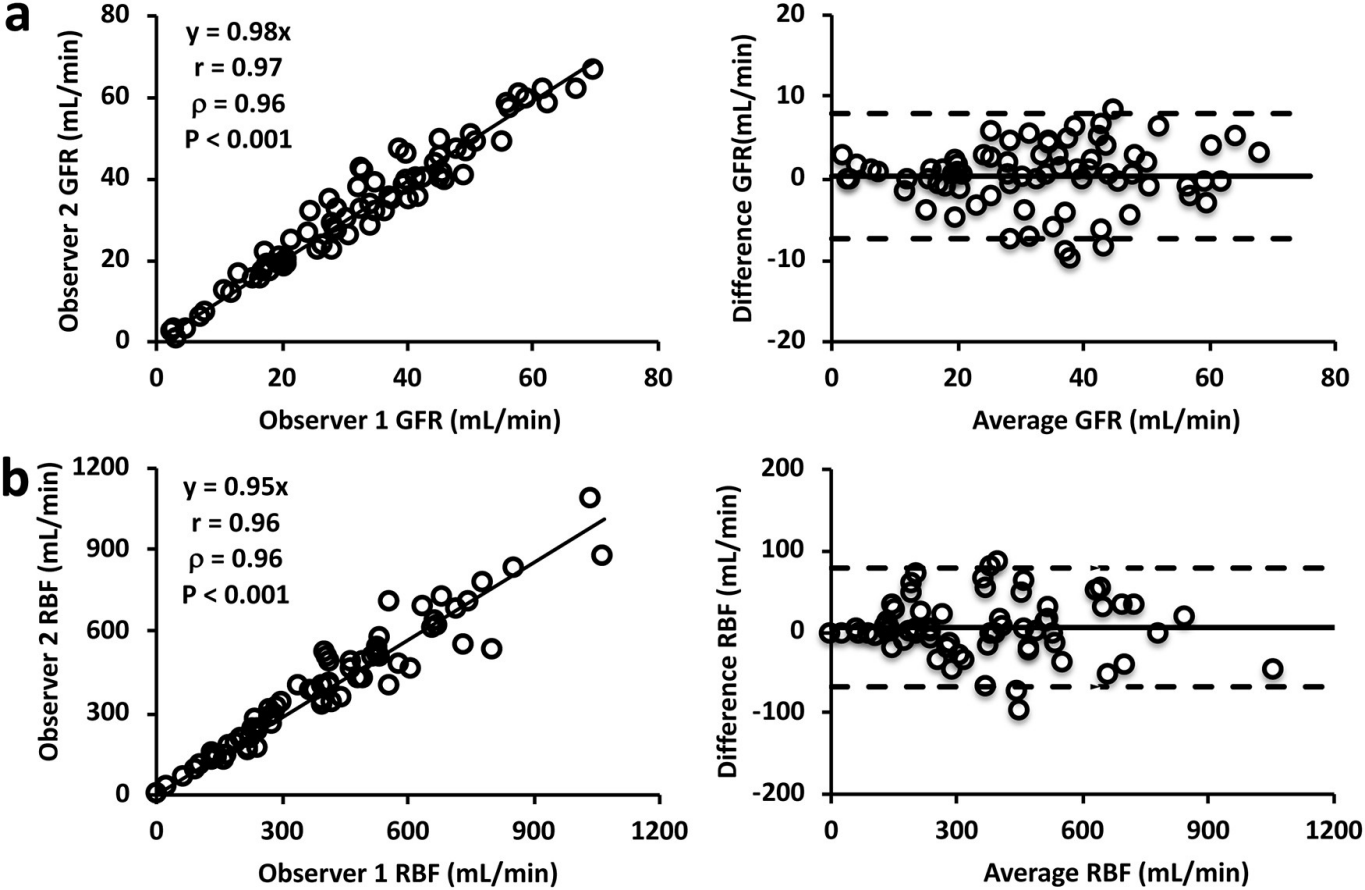
Inter-observer reproducibility of the compartment model in measuring renal functional parameters. (a-b) Correlation (left) and Bland-Altman (right) analyses of the GFR (a) and RBF (b) measured by two independent observers.

The correlation and Bland-Altman analyses showed that inter-observer bias and variation in measurement of the GFR (Fig 5a, r = 0.97, P<0.001; mean difference, 0.3±7.7 mL/min) and RBF (Fig 5b, r = 0.96, P<0.001; mean difference, 5.4±73.1 mL/min) were minimal, suggesting good reproducibility of ROI selection and model fitting.

### Simulation results

**Fig 6:**
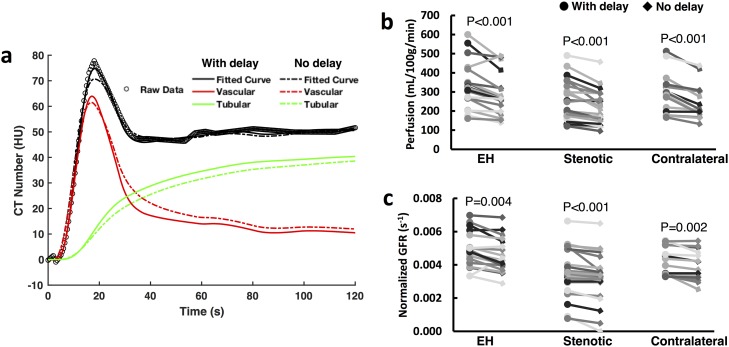
Impact of blood transit delay on estimated renal perfusion and GFR. (a) Representative model fitting of experimentally-acquired data with and without taking into account the blood transit delay from abdominal aorta to kidney. The model-fitted vascular and tubular time attenuation curves are also shown. (b, c) Graphs show changes in model-derived renal perfusion (b) and normalized GFR (c) by neglecting the blood transit delay. Each line represents an individual kidney.

Representative model-derived time attenuation curves in the renal parenchyma (black lines) as well as its vascular (red lines) and tubular (green lines) components with (solid lines) and without (dashed lines) blood transit delay as a model parameter are shown in Fig 6a. Overall, the fitted curve (solid black line) in the renal parenchyma with transit delay provided a better fitting to the experimentally-acquired raw data (circles) compared to the one without transit delay (dashed black line), especially at the vascular peak. Without accounting for the transit delay, the fitted vascular and tubular curves also showed different amplitudes and shapes compared to those fitted with the transit delay. Specifically, the peak amplitude of the vascular curve (dashed red line) was lower, and underestimated the raw data at vascular peak. Because the vascular curve was wider, it also led to a lower tubular curve (dashed green line) in order to match the raw data at tubular phase. As a result, the measured renal perfusion was lower (Fig 6b, EH, 15.0%; stenotic, 13.5%; contralateral, 17.9%) and the normalized GFR underestimated (Fig 6c, EH, 7.2%; stenotic, 8.3%; contralateral, 5.9%) in all kidneys.

The impact of ignoring the blood transit delay T_d_ on estimated renal perfusion and GFR at different fitting ranges and temporal resolutions is shown in S1 and S2 Figs, respectively. In all three simulations, renal perfusion and GFR ratios were smaller than 1.0 at all fitting ranges from 50 to 120 s, indicating that neglecting T_d_ led to an underestimation in renal functional parameters. Similar findings were observed when different temporal resolutions ranging from 0.5 to 3.5 s/scan were used (S2 Fig). Therefore, the necessity of including T_d_ as an unknown parameter in the model fitting for reliable estimation of renal function is independent of the fitting range and temporal resolution.

**Fig 7:**
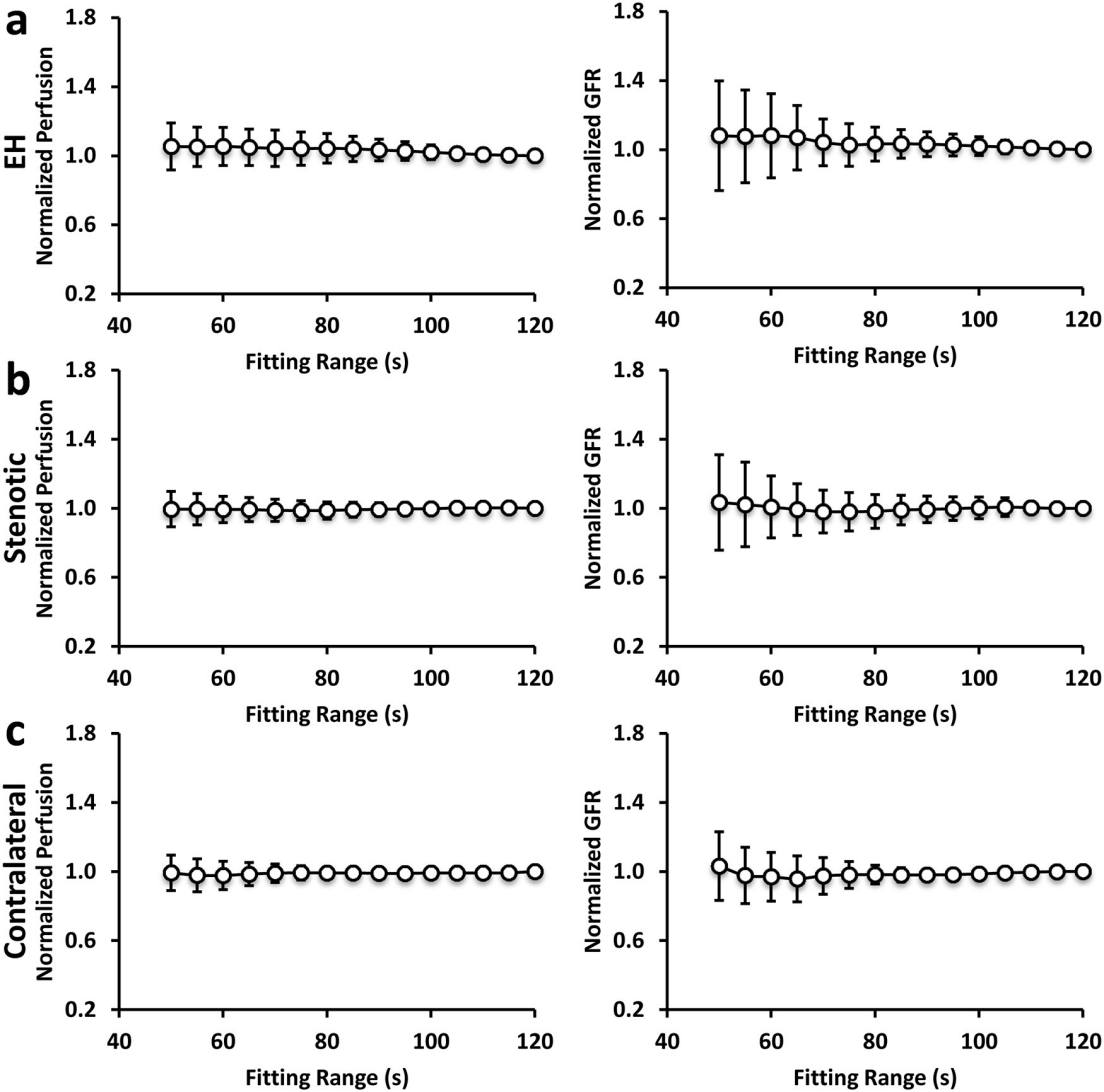
Impact of model fitting range on estimated renal perfusion and GFR. The averaged renal perfusion and normalized GFR as well as their respective standard deviation in EH (a), stenotic (b), and contralateral (c) kidneys with different fitting ranges from 50 to 120 sec. A smaller fitting range led to a larger variation, especially in normalized GFR.

The impact of fitting-range on measurement of perfusion and normalized GFR in EH, stenotic, and contralateral kidneys is shown in Fig 7. The averaged renal perfusion and normalized GFR showed small variations with different fitting ranges from 50 to 120 sec, indicating a robust fitting by the two-compartment model. However, a smaller fitting range led to a larger variation, especially in normalized GFR. In order to maintain a standard deviation under 10%, a minimum fitting range of at least 80 sec seems to be required. This permits elimination of 9 out of 45 scans, thereby decreasing the patient dose by about 20%.

## Discussion

This study applied a modified two-compartment model developed for DCE-MRI for measuring human single-kidney GFR using DCE-CT. The model provided robust fitting to the DCE-CT data and yielded GFR and renal perfusion comparable to those provided by iothalamate clearance and deconvolution, respectively. Good inter-observer reproducibility of GFR and RBF measurement using the proposed model was demonstrated. We also identified decreased renal perfusion, blood flow, and GFR in stenotic kidneys, and demonstrated the importance of accounting for a blood transit delay from the abdominal aorta to the kidney. Further, a CT dynamic imaging of 80 sec was found sufficient for accurate measurement of patient GFR.

Single-kidney GFR measurement is important in evaluating unilateral kidney diseases. Conventional techniques based on serum clearance of endogenous [33] or exogenous [34] markers cannot measure single-kidney GFR, and are limited by collections of urine or blood samples. In contrast, DCE-CT offers a tool for simultaneous assessment of renal anatomy and function with high spatial resolution. It can play an important role in evaluating efficacy of interventions employed to restore RBF in both experimental [35–37] and clinical [38,39] ischemic renal diseases. The importance of this powerful capability is underscored by the advent of novel strategies to target the kidney directly. However, various models proposed for GFR measurement have been only partly successful [3–6,8,9].

Several compartment models have been proposed for measuring GFR using DCE-MRI [10–13], but their application may be hampered by the nonlinearity between MR signal enhancement and gadolinium concentration [40]. Further, the susceptibility effect of gadolinium may decrease MR signal decrease with increasing concentration [41]. A phantom calibration method [42] developed to convert signal intensity to gadolinium concentration could not account for in-vivo signal variations due to B_1_/B_0_ field inhomogeneity at high magnetic fields [43]. Contrarily, the linear relationship between iodinated contrast media and CT number [27] allows accurate measurement of contrast dynamics. In line with this notion, DCE-CT offers lower variability in measured tissue perfusion parameters than DCE-MRI [44]. Therefore, DCE-CT provides a good opportunity to test the conceptual validity of the compartment model in measuring renal functional parameters using DCE-CT. Nevertheless, compared to MRI, DCE-CT involves ionizing radiation dose and iodinated contrast agents, which may cause renal injury in patients with reduced renal function. One needs to evaluate the benefit over risk on a patient-by-patient basis and follow the ALARA principle.

In this study, we applied a modified two-compartment model, previously validated for measurement of murine single-kidney GFR using DCE-MRI [15], for measurements of patient GFR using DCE-CT. This model provided accurate fitting to iodinated time attenuation curves in both EH and ARAS kidneys, indicating that it reliably modeled renal perfusion, filtration, and outflow. Its major advantage over other models is quantification of the contrast outflow curve, hereby inherently accounting for the tubular transit delay. In addition, image analysis is simple and straightforward since no corticomedullary segmentation is needed, as compared to the gamma-variate [8] or three-compartment [13] models. Notably, unlike the compartmental model, the clinically available Baumann–Rudin model [45] is AIF-independent and may therefore be simpler and more reproducible. Nevertheless, it cannot measure renal perfusion, an important renal functional index. Incidentally, CT offers more robust delineation of the AIF than MRI, thanks to its superior signal-to-noise ratio as well as the strict linearity between signal intensity and contrast concentration.

Despite a good correlation with iothalamate clearance, the model-derived GFR was slightly underestimated, for several possible reasons. Both tubular secretion of iothalamate [46,47] and short duration of plasma sampling after administration [48] may overestimate GFR. We also cannot rule out inaccuracies in measurement of iothalamate concentration or physiological changes in GFR in the 2 days between the two measurements. Alternatively, inaccuracies in modeling fitting or acute effects of contrast media may also undermine CT-derived GFR. Nevertheless, the DCE-CT measured GFR was close to the iothalamate GFR, indicating the validity of the model in GFR estimation. Variation within 10% of GFR is generally considered acceptable [49]. Compared to other models, including the Patlak model [3,4,9], gamma-variate model [8,9] and three-compartment model [13], our model has shown superior accuracy in GFR estimation by DCE-CT. Additionally, our model simultaneously provides reliable measurement of renal perfusion, another sensitive biomarker of early-stage kidney disease [50].

Accounting for blood transit delay from aorta to kidney has been inconsistent in kinetic modeling [10,11,13,14]. This study demonstrates the importance of accounting for this delay in human kidneys. Blood velocity in abdominal aorta changes following cardiac cycle [24]. The blood transit delay can be safely ignored in rodents due to their fast heart rate [15], but not in large animals and humans. Indeed, we have shown that neglecting this delay caused sub-optimal fitting to the contrast dynamics and underestimated GFR. Similar sub-optimal curve fitting in the vascular phase has been shown in previous studies [11,51], underscoring the need to employ such a delay in the compartment model for reliable measurement of renal function.

There are several other limitations in our study. First, the model-derived renal perfusion was only compared to that measured using the deconvolution method, but not validated against other reference methods. Nonetheless, this has already been demonstrated in murine kidneys [15]. Second, we only validated this model in EH and ARAS patients. Its accuracy in other forms of kidney diseases remains to be investigated. In addition, only four slices were imaged during DCE-CT as a tradeoff between spatial coverage and temporal resolution, and one mid-hilar representative slice analyzed. Whether volumetric or multislice imaging/analysis would facilitate evaluating patchy renal function in kidneys with focal cysts or lesions remains to be investigated. Third, we used a catheter in the right atrium for a contrast media bolus injection at a relatively fast rate, in order to minimize bolus dispersion. Nevertheless, less invasive and slower peripheral venous injections may be equally effective and useful [52]. Further, a comparison between our model and other established models [53] may better justify its accuracy in measuring renal function, but such data was unavailable in the current study. Moreover, future studies are still needed to test the utility of the compartment model in monitoring renal disease progression or renal recovery after therapeutic treatment.

In conclusion, the modified two-compartment model provided accurate measurement of single-kidney GFR in patients using DCE-CT. This model may offer a useful tool for evaluation of kidney function and assessment of therapeutic interventions using both CT and MRI.

## Supporting information

S1 Dataset Raw data necessary to reproduce our results in this study. (XLSX)

S1 Fig Impact of the blood transit delay T_d_ on estimated renal perfusion and GFR ratios at different fitting ranges. Perfusion (a) and GFR (b) ratios in EH, stenotic, and contralateral kidneys with different fitting ranges from 50 to 120 sec with 5-sec incremental steps. The ratio is calculated as a normalization of the estimated renal parameters without T_d_ as an unknown parameter by those with T_d_ as an unknown parameter. Regardless of fitting ranges, ignoring T_d_ leads to underestimated renal perfusion and GFR.
(TIF)

S2 Fig Impact of the blood transit delay T_d_ on estimated renal perfusion and GFR ratios at different imaging temporal resolutions. Perfusion (a) and GFR (b) ratios in EH, stenotic, and contralateral kidneys with different temporal resolutions from 0.5 to 3.5 sec/scan with 0.2-sec/scan incremental steps. The ratio is calculated as a normalization of the estimated renal parameters without T_d_ as an unknown parameter by those with T_d_ as an unknown parameter. Regardless of temporal resolutions, ignoring T_d_ leads to similar degrees of underestimation in renal perfusion and GFR.
(TIF)
