# Supplementary figures and images for: A modified two-compartment model for measurement of renal function using dynamic contrast-enhanced computed tomography
Source: PLoS One. 2019 Jul 10;14(7):e0219605. doi: 10.1371/journal.pone.0219605 (PMC6619810; doi:10.1371/journal.pone.0219605)

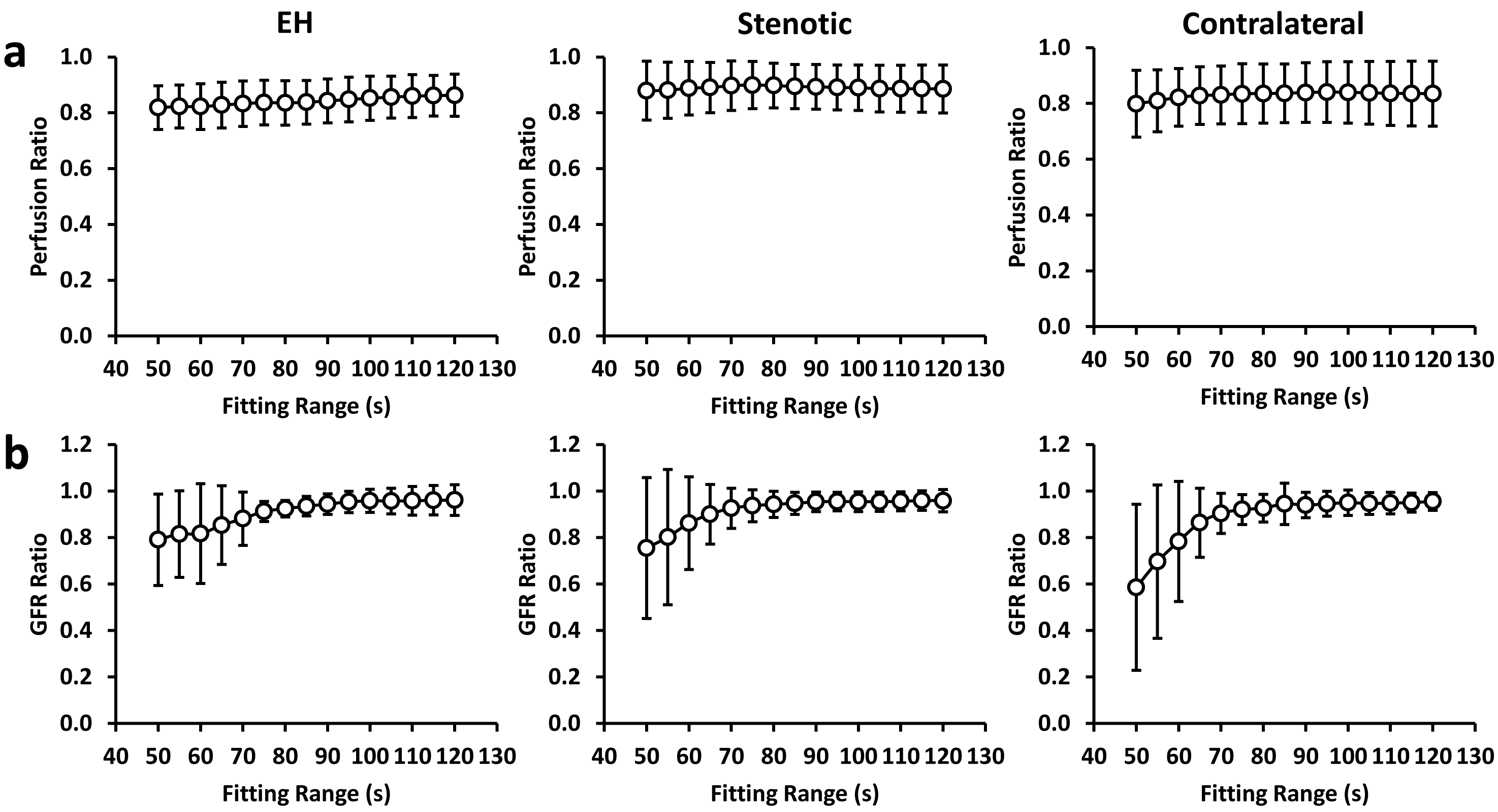

Supplement: S1 Fig — Perfusion (a) and GFR (b) ratios in EH, stenotic, and contralateral kidneys with different fitting ranges from 50 to 120 sec with 5-sec incremental steps. The ratio is calculated as a normalization of the estimated renal parameters without Td as an unknown parameter by those with Td as an unknown parameter. Regardless of fitting ranges, ignoring Td leads to underestimated renal perfusion and GFR. (TIF) [file pone.0219605.s002.tif]

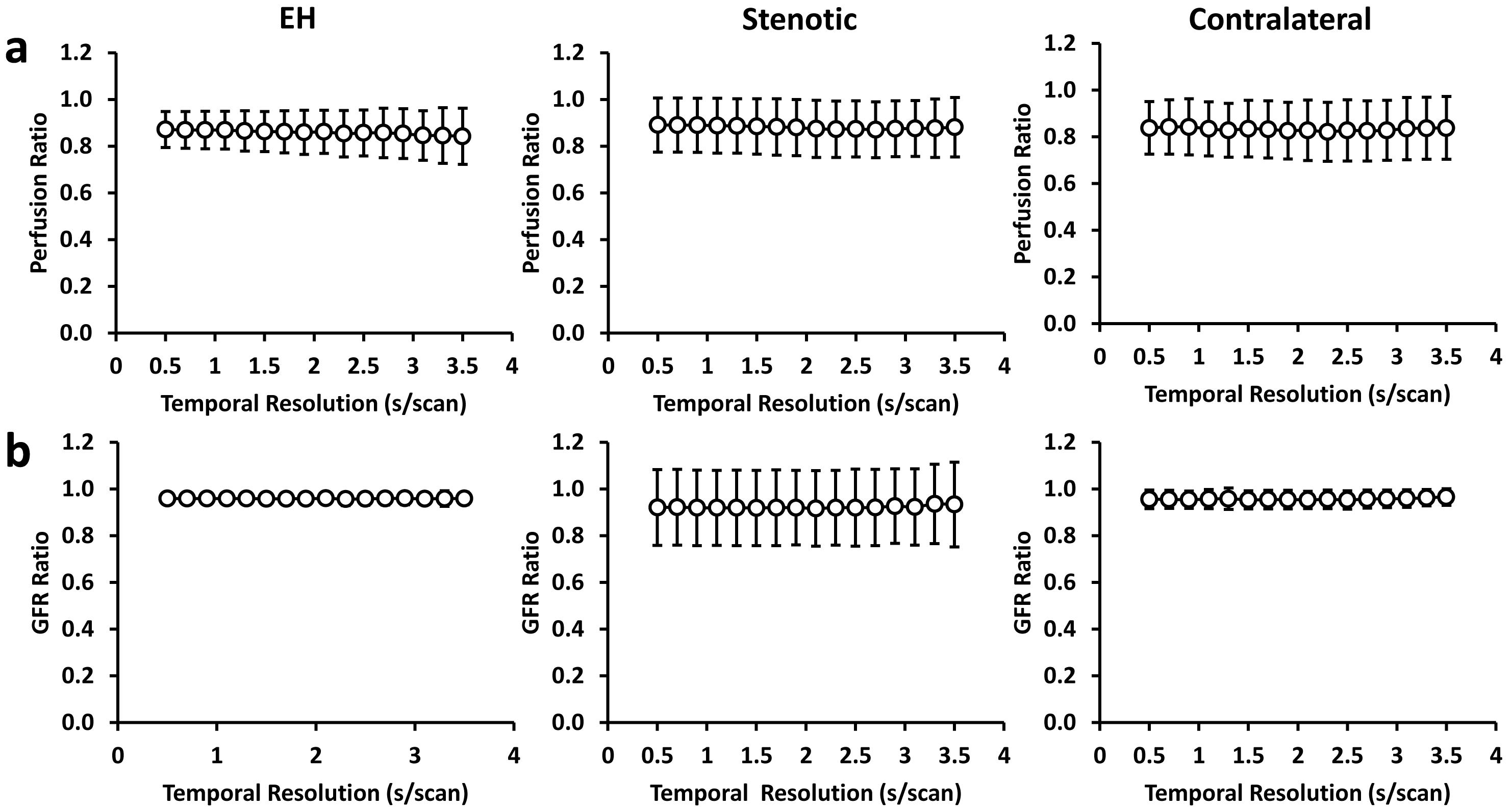

Supplement: S2 Fig — Perfusion (a) and GFR (b) ratios in EH, stenotic, and contralateral kidneys with different temporal resolutions from 0.5 to 3.5 sec/scan with 0.2-sec/scan incremental steps. The ratio is calculated as a normalization of the estimated renal parameters without Td as an unknown parameter by those with Td as an unknown parameter. Regardless of temporal resolutions, ignoring Td leads to similar degrees of underestimation in renal perfusion and GFR. (TIF) [file pone.0219605.s003.tif]
